# A Hybrid SSA and SMA with Mutation Opposition-Based Learning for Constrained Engineering Problems

**DOI:** 10.1155/2021/6379469

**Published:** 2021-09-07

**Authors:** Shuang Wang, Qingxin Liu, Yuxiang Liu, Heming Jia, Laith Abualigah, Rong Zheng, Di Wu

**Affiliations:** ^1^School of Information Engineering, Sanming University, Sanming 365004, China; ^2^School of Computer Science and Technology, Hainan University, Haikou 570228, China; ^3^College of Physics and Information Engineering, Fuzhou University, Fuzhou 350108, China; ^4^Faculty of Computer Sciences and Informatics, Amman Arab University, Amman 11953, Jordan; ^5^School of Computer Science, Universiti Sains Malaysia, Gelugor, Penang, Malaysia; ^6^School of Education and Music, Sanming University, Sanming 365004, China

## Abstract

Based on Salp Swarm Algorithm (SSA) and Slime Mould Algorithm (SMA), a novel hybrid optimization algorithm, named Hybrid Slime Mould Salp Swarm Algorithm (HSMSSA), is proposed to solve constrained engineering problems. SSA can obtain good results in solving some optimization problems. However, it is easy to suffer from local minima and lower density of population. SMA specializes in global exploration and good robustness, but its convergence rate is too slow to find satisfactory solutions efficiently. Thus, in this paper, considering the characteristics and advantages of both the above optimization algorithms, SMA is integrated into the leader position updating equations of SSA, which can share helpful information so that the proposed algorithm can utilize these two algorithms' advantages to enhance global optimization performance. Furthermore, Levy flight is utilized to enhance the exploration ability. It is worth noting that a novel strategy called mutation opposition-based learning is proposed to enhance the performance of the hybrid optimization algorithm on premature convergence avoidance, balance between exploration and exploitation phases, and finding satisfactory global optimum. To evaluate the efficiency of the proposed algorithm, HSMSSA is applied to 23 different benchmark functions of the unimodal and multimodal types. Additionally, five classical constrained engineering problems are utilized to evaluate the proposed technique's practicable abilities. The simulation results show that the HSMSSA method is more competitive and presents more engineering effectiveness for real-world constrained problems than SMA, SSA, and other comparative algorithms. In the end, we also provide some potential areas for future studies such as feature selection and multilevel threshold image segmentation.

## 1. Introduction

In recent years, metaheuristic algorithms have been widely concerned by a large number of scholars. Compared with other traditional optimization algorithms, the concept of metaheuristic algorithms is simple. Besides, they are flexible and can bypass local optima. Thus, metaheuristics have been successfully applied in different fields to solve various complex optimization problems in the real world [1–3].

Metaheuristic algorithms include three main categories: evolution-based, physics-based, and swarm-based techniques. The inspirations of evolutionary-based methods are the laws of evolution in nature. The most popular evolution-based algorithms include Genetic Algorithm (GA) [4], Differential Evolution Algorithm (DE) [5], and Biogeography-Based Optimizer (BBO) [6]. Physics-based algorithms mimic the physical rules in the universe. There are some representative algorithms such as Simulated Annealing (SA) [7], Gravity Search Algorithm (GSA) [8], Black Hole (BH) algorithm [9], Multiverse Optimizer (MVO) [10], Artificial Chemical Reaction Optimization Algorithm (ACROA) [11], Ray Optimization (RO) [12], Curved Space Optimization (CSO) [13], Sine Cosine Algorithm (SCA) [14], Arithmetic Optimization Algorithm (AOA) [15], and Heat Transfer Relation-based Optimization Algorithm (HTOA) [16]. The third category algorithm is swarm-based techniques, which simulate the social behavior of creatures in nature. Some optimization techniques of this class include Particle Swarm Optimization (PSO) [17] Ant Colony Optimization Algorithm (ACO) [18], Firefly Algorithm (FA) [19], Grey Wolf Optimizer (GWO) [20], Cuckoo Search (CS) Algorithm [21], Whale Optimization Algorithm (WOA) [22], Bald Eagle Search (BES) algorithm [23], and Aquila Optimizer (AO) [24].

It is worth noting that the most widely used swarm-based optimization algorithm is PSO [25]. PSO simulates the behavior of birds flying together in flocks. During the search, they all follow the best solutions in their paths. Cacciola et al. [26] discussed the problem of corrosion profile reconstruction starting from electrical data, in which PSO was utilized to obtain the image of the reconstructed corrosion profile. The result shows that PSO can obtain the optimal solution compared with LSM, and it takes the least time. This allows us to recognize the huge potential of the optimization algorithm.

Salp Swarm Algorithm (SSA) [27] is a swarm-based algorithm proposed in 2017. SSA is inspired by swarm behavior, navigation, and foraging of salps in the ocean. Since SSA has fewer parameters and is easier to be realized in a program than other algorithms, SSA has been applied to many optimization problems, such as feature selection, image segmentation, and constrained engineering problems. However, like other metaheuristic algorithms, SSA may be easy to trap into local minima and lower population density. Therefore, many improved researches have been proposed to enhance the performance of SSA in many fields. Tubishat et al. [28] presented a Dynamic SSA (DSSA), which shows better accuracy than SSA in feature selection. Salgotra et al. [29] proposed a self-adaptive SSA to enhance exploitation ability and convergence speed. Neggaz et al. [30] proposed an improved leader in SSA using Sine Cosine Algorithm and disrupt operator for feature selection. Jia and Lang [31] presented an enhanced SSA with a crossover scheme and Levy flight to improve the movement patterns of salp leaders and followers. There are also other attempts on the hybrid algorithm of SSA. Saafan and El-gendy [32] proposed a hybrid improved Whale Optimization Salp Swarm Algorithm (IWOSSA). The IWOSSA achieves a better balance between exploration and exploitation phases and avoids premature convergence effectively. Singh et al. [33] developed a hybrid SSA with PSO, which integrated the advantages of SSA and PSO to eliminate trapping in local optima and unbalanced exploitation. Abadi et al. [34] proposed a hybrid approach by combining SSA with GA, which could obtain good results in solving some optimization problems.

Slime Mould Algorithm (SMA) [35] is the latest swarm intelligence algorithm proposed in 2020. This algorithm simulates the oscillation mode and the foraging of Slime Mould in nature. SMA has a unique search mode, which keeps the algorithm from falling into local optima, and has superior global exploration capability. The approach has been applied in real-world optimization problems like feature selection [36], parameters optimization of the fuzzy system [37], multilevel threshold image segmentation [38], control scheme [39], and parallel connected multistacks fuel cells [40].

Therefore, based on the capabilities of both above algorithms, we try to do a hybrid operation to improve the performance of SMA or SSA and then propose a new hybrid optimization algorithm (HSMSSA) to speed up the convergence rate and enhance the overall optimization performance. The specific method is that we integrate SMA as the leader role into SSA and retain the exploitation phase of SSA. At the same time, inspired by the significant performance of opposition-based learning and quasiopposition-based learning, we propose a new strategy named mutation opposition-based learning (MOBL), which switches the algorithm between opposition-based learning and quasiopposition-based learning through a mutation rate to increase the diversity of the population and speed up the convergence rate. In addition, Levy flight is utilized to improve SMA's exploration capability and balance the exploration and exploitation phases of the algorithm. The proposed HSMSSA algorithm can improve both the exploration and exploitation abilities. The proposed HSMSSA is tested on 23 different benchmark functions and compared with other optimization algorithms. Furthermore, five constrained engineering problems are also utilized to evaluate HSMSSA's capability on real-world optimization problems. The experimental results illustrate that the HSMSSA possesses the superior capability to search the global minimum and achieve less cost engineering design results than other state-of-the-art metaheuristic algorithms.

The remainder of this paper is organized as follows.  Section 2 provides a brief overview of SSA, SMA, Levy flight, and mutation opposition-based learning strategy.  Section 3 describes the proposed hybrid algorithm in detail. In Section 4, the details of benchmark functions, parameter settings of the selected algorithms, simulation experiments, and results analysis are introduced. Conclusions and prospects are given in Section 5.

## 2. Preliminaries

### 2.1. Salp Swarm Algorithm

**Figure 1:**
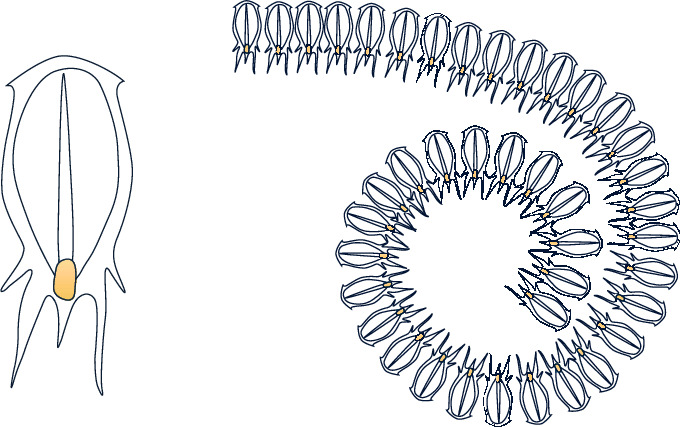
Individual salp and the swarm of salp [25].

In the deep sea, salps live in groups and form a salp chain to move and forage. In the salp chain, there are leaders and followers. The leader moves towards the food and guides the followers. In the process of moving, leaders explore globally, while followers thoroughly search locally [27]. The shapes of a salp and salp chain are shown in Figure 1.

#### 2.1.1. Leader Salps

The front salp of the chain is called the leader, so the following equation is used to perform this action to the salp leader:

(1) $$\begin{array}{c} X_{j}^{1}=\left\{\begin{array}{cc} F_{j}+c_{1}\left(\left(\text{UB}-\text{LB}\right)r_{1}+\text{LB}\right), & r_{2}<0.5,\\ F_{j}-c_{1}\left(\left(\text{UB}-\text{LB}\right)r_{1}+\text{LB}\right), & r_{2}\geq 0.5, \end{array}\right. \end{array}$$

(2) $$\begin{array}{c} c_{1}=2e^{-{\left(4t/T\right)}^{2}}, \end{array}$$

where *X*_*j*_^1^ and *F*_*j*_ represent the new position of the leader and food source in the *j*th dimension and *r*_1_ and *r*_2_ are randomly generated numbers in the interval [0, 1]. It is worth noting that *c*_1_ is essential for SSA because it balances exploration and exploitation during the search process. *t* is the current iteration and *T* is the max iteration.

#### 2.1.2. Follower Salps

To update the position of the followers, the new concept is introduced, which is based on Newton's law of motion as in the following equation:

(3) $$\begin{array}{c} X_{j}^{i}=\frac{1}{2}gt^{2}+\omega_{0}t,\;i\geq 2, \end{array}$$

where *X*_*j*_^*i*^ represents the position of *i*th follower salp in the *j*th dimension and *g* and *ω*_0_ indicate the acceleration and the velocity, respectively. The updating process of followers can be expressed as in the following equation:

(4) $$\begin{array}{c} X_{j}^{i}=\frac{1}{2}\left(X_{j}^{i}+X_{j}^{i-1}\right). \end{array}$$

The pseudocode of SSA is presented in Algorithm 1.

### 2.2. Slime Mould Algorithm

The main idea of SMA is inspired by the behavior and morphological changes of Slime Mould in foraging. Slime Mould can dynamically change search mode based on the quality of food sources. If the food source has a high quality, the Slime Mould will use the region-limited search method. If the food concentration is low, the Slime Mould will explore other food sources in the search space. Furthermore, even if Slime Mould has found a high-quality food source, it still divides some individuals to explore another area in the region [35]. The behavior of Slime Mould can be mathematically described as follows:

(5) $$\begin{array}{c} \overset{⟶}{X\left(t+1\right)}=\left\{\begin{array}{cc} r_{4}\times \left(\text{UB}-\text{LB}\right)+\text{LB}, & r_{3}<z,\\ \overset{⟶}{X_{b}\left(t\right)}+\overset{⟶}{vb}\times \left(\overset{⟶}{W}\cdot \overset{⟶}{X_{A}\left(t\right)}-\overset{⟶}{X_{B}\left(t\right)}\right), & r_{5}<p,\\ \overset{⟶}{vc}\times \overset{⟶}{X\left(t\right)}, & r_{5}\geq p, \end{array}\right. \end{array}$$

where parameters *r*_3_, *r*_4_, and *r*_5_ are random values in the range of 0 to 1. *UB* and *LB* indicate the upper and lower bound of search space. *z* is a constant. $\overset{⟶}{X_{b}\left(t\right)}$ represents the best position obtained in all iterations, $\overset{⟶}{X_{A}\left(t\right)}$ and $\overset{⟶}{X_{B}\left(t\right)}$ represent two individuals selected randomly from the population, and $\overset{⟶}{X\left(t\right)}$ represents the location of Slime Mould. $\overset{⟶}{vc}$ decreases linearly from one to zero, and $\overset{⟶}{vb}$ is an oscillation parameter in the range [−*a*, *a*], in which *a* is calculated as follows:

(6) $$\begin{array}{c} a=\arctan\;h\left(-\left(\frac{t}{T}\right)+1\right). \end{array}$$

The coefficient $\overset{⟶}{W}$ is a very important parameter, which simulates the oscillation frequency of different food concentrations so that Slime Mould can approach food more quickly when they find high-quality food. The formula of $\overset{⟶}{W}$ is listed as follows:

(7) $$\begin{array}{c} \overset{⟶}{W\left(\text{SmellIndex}\left(i\right)\right)}=\left\{\begin{array}{cc} 1+r_{6}\times \;\log\left(\frac{bF-S\left(i\right)}{bF-wF}+1\right), & \text{condition},\\ 1-r_{6}\times \;\log\left(\frac{bF-S\left(i\right)}{bF-wF}+1\right), & \text{others}, \end{array}\right. \end{array}$$

(8) $$\begin{array}{c} \text{SmellIndex}=\text{sort}\left(S\right), \end{array}$$

where *i* ∈ 1, 2,…, *N* and *S*(*i*) represents the fitness of *X*. *condition* indicates that *S*(*i*) ranks first half of the Slime Mould, and *r*_6_ are random numbers uniformly generated in the interval of [0, 1]. *bF* represents the optimal fitness obtained in the current iterative process, *wF* represents the worst fitness value obtained in the iterative process currently, and *SmellIndex* denotes the sequence of fitness values sorted (ascends in the minimum value problem).

**Figure 2:**
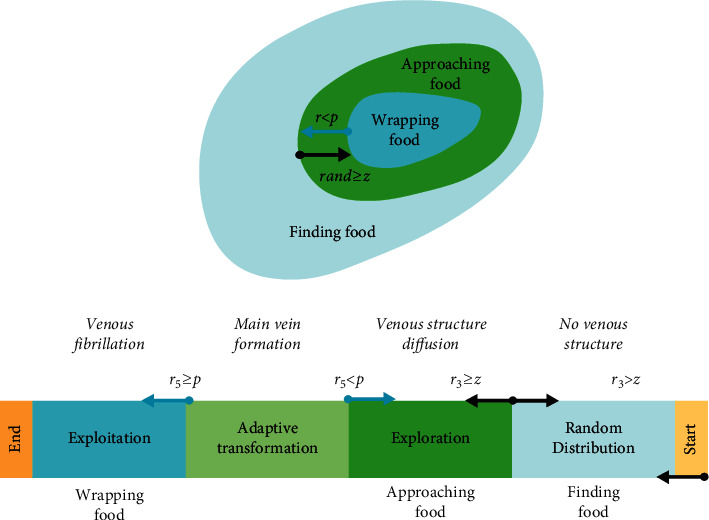
The steps of SMA.

The *p* parameter can be described as follows:

(9) $$\begin{array}{c} p=\tanh\left|S\left(i\right)-\text{DF}\right|, \end{array}$$

where DF represents the best fitness over all iterations.  Figure 2 visualizes the general logic of SMA.

The pseudocode of SMA is presented in Algorithm 2.

### 2.3. Levy Flight

**Figure 3:**
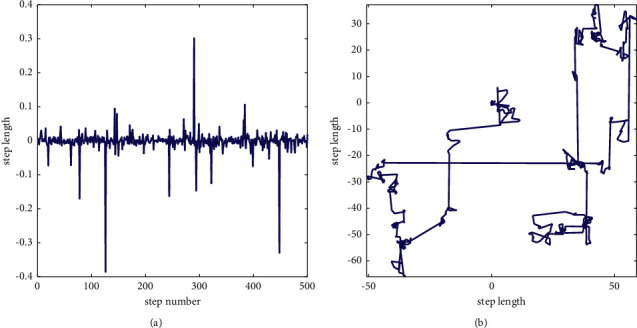
Levy distribution and 2D Levy trajectory.

Levy flight is an effective strategy for metaheuristic algorithms, successfully designed in many algorithms [41–44]. Levy flight is a class of non-Gaussian random processes that follows Levy distribution. It alternates between short-distance and occasionally long-distance walking, which can be inferred from Figure 3. The formula of Levy flight is as follows:

(10) $$\begin{array}{c} \text{Levy}=0.01\times \frac{r_{7}\times \sigma }{{\left|r_{8}\right|}^{\left(1/\beta \right)}},\\ \sigma ={\left(\frac{\Gamma \left(1+\beta \right)\times \;\sin\left(\pi \beta /2\right)}{\Gamma \left(\left(1+\beta \right)/2\right)\times \beta \times 2^{\left(\left(\beta -1\right)/2\right)}}\right)}^{\left(1/\beta \right)}, \end{array}$$

where *r*_7_ and *r*_8_ are random values in the range of [0, 1] and *β* is a constant equal to 1.5.

### 2.4. Mutation Opposition-Based Learning

Opposition-based learning (OBL) was proposed by Tizhoosh in 2005 [45]. The essence of OBL is selecting the best solution to the next iteration by comparing the current solution and its opposition-based learning solution. The OBL strategy has been successfully used in varieties of metaheuristic algorithms [46–51] to improve the ability of local optima stagnation avoidance, and the mathematical expression is as follows:

(11) $$\begin{array}{c} X_{\text{OBL}}\left(t+1\right)=\text{LB}+\text{UB}-X\left(t\right). \end{array}$$

Quasiopposition-based learning (QOBL) [52] is an improved version from OBL, which applies quasiopposite points instead of opposite points. These points produced through QOBL have more likelihood of being unknown solutions than the points created by OBL. The mathematical formula of QOBL is as follows:

(12) $$\begin{array}{c} X_{\text{QOBL}}\left(t+1\right)=\left\{\begin{array}{cc} \text{CS}+r_{9}\times \left(\text{MP}-\text{CS}\right), & \text{if MP}>\text{CS},\\ \text{MP}+r_{9}\times \left(\text{CS}-\text{MP}\right), & \text{otherwise,} \end{array}\right.\\ \text{CS}=\frac{\text{LB}+\text{UB}}{2},\\ \text{MP}=\text{LB}+\text{UB}-X\left(t\right). \end{array}$$

**Figure 4:**
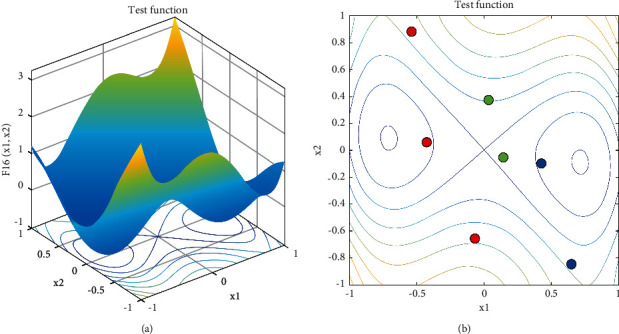
MOBL example: (a) objective function and (b) candidate solutions (red point), its OBL solution (blue point), and its QOBL solution (green point).

Considering the superior performance of the two kinds of opposition-based learning, we propose mutation opposition-based learning (MOBL) by combining the mutation rate with these two opposition-based learning. By selecting the mutation rate, we can give full play to the characteristics of the OBL and QOBL and effectively enhance the ability of the algorithm to jump out of the local optima.  Figure 4 is an MOBL example, in which Figure 4(a) shows an objective function and Figure 4(b) displays three candidate solutions and their OBL solutions or QOBL solutions. The mathematical formula is as follows:

(13) $$\begin{array}{c} X\left(t+1\right)=\left\{\begin{array}{cc} X_{\text{OBL}}\left(t\right), & if\;r_{10}<\text{rate},\\ X_{\text{QOBL}}\left(t\right), & \text{else,} \end{array}\right. \end{array}$$

where *rate* is mutation rate, and we set it to 0.1.

## 3. The Proposed Algorithm

### 3.1. Details of HSMSSA

In SSA, the population is divided into leader salps and follower salps: leader salps are the first half of salps in the chain, and follower salps follow the leader. However, the leader salp has poor randomness and is easy to fall into local optima. For the SMA algorithm, Slime Mould selects different search modes according to the positive and negative feedback of the current food concentration and has a certain probability of isolating some individuals to explore other regions in search space. These mechanisms increase the randomness of Slime Mould and enhance the ability to explore. The *vb* parameter is utilized to realize the oscillation mode of Slime Mould, which is in the range of [−a, a]. However, *vb* has the drawback of low randomness, which cannot effectively simulate the process of Slime Mould looking for food sources. Therefore, we introduce Levy flight into the exploration phase to further enhance the exploration ability. Next, we integrate SMA into SSA, change the position update method of leader salps, and further improve the randomness of the algorithm through Levy flight. For followers, we propose a mutation opposition-based learning to enhance its population diversity and increase the ability of the algorithm to jump out of the local optima. The mathematical formula of leader salps is as follows:

(14) $$\begin{array}{c} \overset{⟶}{X\left(t+1\right)}=\left\{\begin{array}{cc} r_{4}\times \left(\text{UB}-\text{LB}\right)+\text{LB}, & r_{3}<z,\\ X_{b}+vb\times \left(W\times X_{A}-X_{B}\right)\times \text{Levy}, & r_{5}<p,\\ vc\times X_{i}, & r_{5}\geq p. \end{array}\right. \end{array}$$

**Figure 5:**
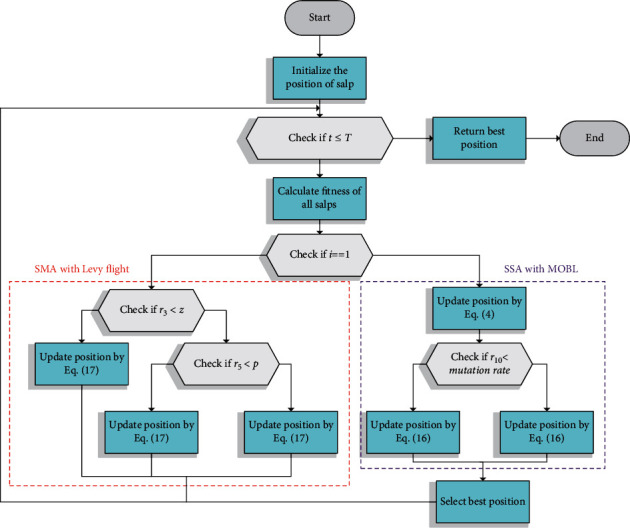
Flowchart of HSMSSA.

The pseudocode of HSMSSA is given in Algorithm 3, and the summarized flowchart is displayed in Figure 5. As shown in Algorithm 3, the position of the population is initially generated randomly. Then, each individual's fitness will be calculated. For the entire population in each iteration, parameter W is calculated using equation (7). The search agents of population size N are assigned to the two algorithms, which can utilize the advantages of SSA and SMA, and realize the sharing of helpful information to achieve global optimization. If the search agent belongs to the first half of the population, the position will be updated using equation (14) in SMA with Levy flight. Otherwise, the position is determined using equation (4) and MOBL. Finally, if the termination criteria are satisfied, the algorithm returns the best solution found so far; else the previous steps are repeated.

### 3.2. Computational Complexity Analysis

HSMSSA mainly consists of three components: initialization, fitness evaluation, and position updating. In the initialization phase, the computational complexity of positions generated is O(*N* × *D*), where *D* is the dimension size of the problem. Then, the computational complexity of fitness evaluation for the solution is O(N) during the iteration process. Finally, we utilize mutation opposition-based learning to keep the algorithm from falling into local optima; thus, the computational complexities of position updating of HSMSSA are O(2 × *N* × *D*). Therefore, the total computational complexity of the proposed HSMSSA algorithm is O(*N* × *D* + *N* + 2 × *N* × *D*).

## 4. Experimental Results and Discussion

This section compared the HSMSSA with some state-of-the-art metaheuristics algorithms on 23 benchmark functions to validate its performance. Moreover, five engineering design problems are employed as examples for real-world applications. The experimentations ran on Windows 10 with 24 GB RAM and Intel (R) i5-9500. All simulations were carried out using MATLAB R2020b.

### 4.1. Definition of 23 Benchmark Functions

To assess HSMSSA's ability of exploration, exploitation, and escaping from local optima, 23 benchmark functions, including unimodal and multimodal functions, are tested [27]. The unimodal benchmark functions (F1–F7) are utilized to examine the exploitation ability of HSMSSA. The description of the unimodal benchmark function is shown in Table 1. The multimodal and fixed-dimension multimodal benchmark functions (F8–F23) shown in Tables 2 and 3 are used to test the exploration ability of HSMSSA.

In order to show the experimental results more representative, the HSMSSA is compared with the basic SMA [35] and SSA [27], AO [24], AOA [15], WOA [22], SCA [14], and MVO [10]. For all tests, we set the population size *N* = 30, dimension size *D* = 30, and maximum iteration *T* = 500, respectively, for all algorithms with 30 independent runs. The parameter settings of each algorithm are shown in Table 4. After all, average results and standard deviations are employed to evaluate the results. Note that the best results will be bolded.

#### 4.1.1. Evaluation of Exploitation Capability (F1–F7)

As we can see, unimodal benchmark functions have only one global optimum. These functions are allowed to evaluate the exploitation ability of the metaheuristic algorithms. It can be seen from Table 5 that HSMSSA is very competitive with SMA, SSA, and other metaheuristic algorithms. In particular, HSMSSA can achieve much better results than other metaheuristic algorithms except F6. For F1–F4, HSMSSA can find the theoretical optimum. For all unimodal functions except F5, HSMSSA gets the smallest average values and standard deviations compared to other algorithms, which indicate the best accuracy and stability. Hence, the exploitation capability of the proposed HSMSSA algorithm is excellent.

#### 4.1.2. Evaluation of Exploration Capability (F8–F23)

Unlike unimodal functions, multimodal functions have many local optima. Thus, this kind of test problem turns very useful to evaluate the exploration capability of an optimization algorithm. The results shown in Table 5 for functions F8–F23 indicate that HSMSSA also has an excellent exploration capability. In fact, we can see that HSMSSA can find the theoretical optimum in F9, F11, F16–F17, and F19–F23. These results reveal that HSMSSA can also provide superior exploration capability.

#### 4.1.3. Analysis of Convergence Behavior

**Figure 6:**
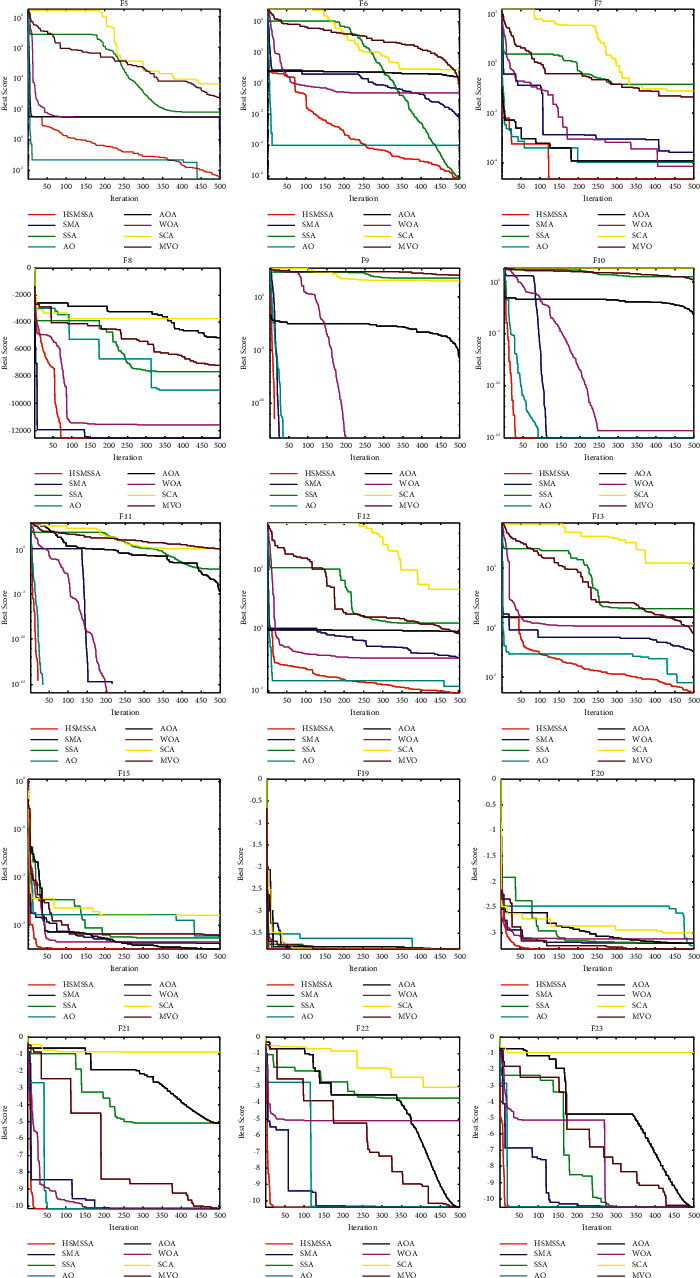
Convergence curves of 23 benchmark functions.

The convergence curves of some functions are selected and shown in Figure 6, which show the convergence rate of algorithms. It can be seen that HSMSSA shows competitive performance compared to other state-of-the-art algorithms. The HSMSSA presents a faster convergence speed than all other algorithms in F7–F13, F15, and F19–F23. For other benchmark functions, HSMSSA shows a better capability of local optima avoidance than other comparison algorithms in F5 and F6.

#### 4.1.4. Qualitative Results and Analysis

**Figure 7:**
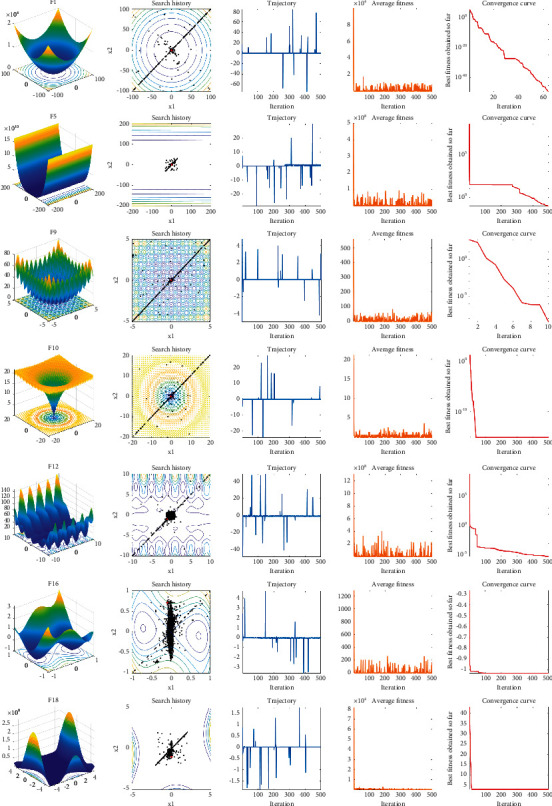
Parameter space, search history, trajectory, average fitness, and convergence curves of HSMSSA.

Furthermore, Figure 7 shows the results of several representative test functions on search history, trajectory, average fitness, and convergence curve. From search history maps, we can see the search agent's distribution of the HSMSSA while exploring and exploiting the search space. Because of the fast convergence, the vast majority of search agents are concentrated near the global optimum. Inspecting trajectory figure in Figure 5, the first search agent constantly oscillates in the first dimension of the search space, which suggests that the search agent investigates the most promising areas and better solutions widely. This powerful search capability is likely to come from the Levy flight and MOBL strategies. The average fitness presents if exploration and exploitation are conducive to improve the first random population, and an accurate approximation of the global optimum can be found in the end.

Similarly, it can be noticed that the average fitness oscillates in the early iterations and then decreases abruptly and begins to level off. The average fitness maps also show the significant improvement of the first random population and the final global optimal, accurate approximation acquisition. At last, the convergence curves reveal the best fitness value found by search agents after each iteration. By observing this, the HSMSSA shows breakneck convergence speed.

#### 4.1.5. Wilcoxon Signed-Rank Test

Because the algorithm results are random, we need to carry out statistical tests to prove that the results have statistical significance. We use Wilcoxon signed-ranks (WSR) test results to evaluate the statistical significance of the two algorithms at 5% significance level [53]. The WSR is a statistical test that is applied to two different results for searching the significantly different. As is well-known, a *p*-value less than 0.05 indicates that it is significantly superior to other algorithms. Otherwise, the obtained results are not statistically significant. The calculated results of the Wilcoxon signed-rank test between HSMSSA and other algorithms for each benchmark function are listed in Table 6. HSMSSA outperforms all other algorithms in varying degrees. This superiority is statistically significant on unimodal functions F2 and F4–F7, which indicates that HSMSSA possesses high exploitation. HSMSSA also shows better results on multimodal function F8–F23, suggesting that HSMSSA has a high capability of exploration. To sum up, HSMSSA can provide better results for almost all benchmark functions than other comparative algorithms.

### 4.2. Experiments on Engineering Design Problems

In this section, HSMSSA is evaluated to solve five classical engineering design problems: pressure vessel design problem, tension spring design problem, three-bar truss design problem, speed reducer problem, and cantilever beam design. To address these problems, we set the population size *N* = 30 and maximum iteration *T* = 500. The results of HSMSSA are compared to various state-of-the-art algorithms in the literature. The parameter settings are the same as previous numerical experiments.

#### 4.2.1. Pressure Vessel Design Problem

**Figure 8:**
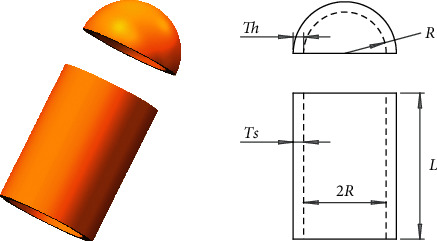
Pressure vessel design problem.

The pressure vessel design problem [53] is to minimize the total cost of cylindrical pressure vessel to match pressure requirements and form the pressure vessel shown in Figure 8. Four parameters in this problem need to be minimized, including the thickness of the shell (Ts), the thickness of head (Th), inner radius (R), and the length of the cylindrical section without the head (L), as shown in Figure 8. The constraints and equation are as follows.

Consider

(15) $$\begin{array}{c} \overset{⟶}{x}=\left[\begin{array}{cccc} x_{1} & x_{2} & x_{3} & x_{4} \end{array}\right]=\left[\begin{array}{cccc} T_{s} & T_{h} & R & L \end{array}\right]. \end{array}$$

Minimize

(16) $$\begin{array}{c} f\left(\overset{⟶}{x}\right)=0.6224x_{1}x_{3}x_{4}+1.7781x_{2}x_{3}^{2}+3.1661x_{1}^{2}x_{4}+19.84x_{1}^{2}x_{3}, \end{array}$$

subject to

(17) $$\begin{array}{c} \left\{\begin{array}{c} g_{1}\left(\overset{⟶}{x}\right)=-x_{1}+0.0193x_{3}\leq 0,\\ g_{2}\left(\overset{⟶}{x}\right)=-x_{3}+0.00954x_{3}\leq 0,\\ g_{3}\left(\overset{⟶}{x}\right)=-\pi x_{3}^{2}x_{4}-\frac{4}{3}\pi x_{3}^{3}+1296000\leq 0,\\ g_{4}\left(\overset{⟶}{x}\right)=x_{4}-240\leq 0. \end{array}\right. \end{array}$$

Variable range is

(18) $$\begin{array}{c} \left\{\begin{array}{c} 0\leq x_{1}\leq 99,\\ 0\leq x_{2}\leq 99,\\ 10\leq x_{3}\leq 200,\\ 10\leq x_{4}\leq 200. \end{array}\right. \end{array}$$

From the results in Table 7, we can see that HSMSSA can obtain superior optimal values compared with SMA, SSA, AO, AOA, WOA, SCA, and MVO.

#### 4.2.2. Tension Spring Design Problem

**Figure 9:**
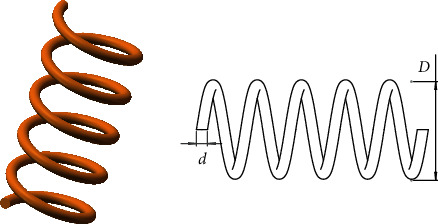
Tension spring design problem.

This problem [27] tries to minimize the weight of the tension spring, and there are three parameters that need to be minimized, including the wire diameter (d), mean coil diameter (D), and the number of active coils (N).  Figure 9 shows the structure of the tension spring. The mathematical of this problem can be written as follows.

Consider

(19) $$\begin{array}{c} \overset{⟶}{x}=\left[\begin{array}{cccc} x_{1} & x_{2} & x_{3} & x_{4} \end{array}\right]=\left[\begin{array}{ccc} d & D & N \end{array}\right]. \end{array}$$

Minimize

(20) $$\begin{array}{c} f\left(\overset{⟶}{x}\right)=\left(x_{3}+2\right)x_{2}x_{1}^{2}, \end{array}$$

subject to

(21) $$\begin{array}{c} g_{1}\left(\overset{⟶}{x}\right)=1-\frac{x_{2}^{3}x_{3}}{71785x_{1}^{4}}\leq 0,\\ g_{2}\left(\overset{⟶}{x}\right)=\frac{4x_{2}^{2}-x_{1}x_{2}}{12566\left(x_{2}x_{1}^{3}-x_{1}^{4}\right)}+\frac{1}{5108x_{1}^{2}}\leq 0,\\ g_{3}\left(\overset{⟶}{x}\right)=1-\frac{140.45x_{1}}{x_{2}^{2}x_{3}}\leq 0,\\ g_{4}\left(\overset{⟶}{x}\right)=\frac{x_{1}+x_{2}}{1.5}-1\leq 0. \end{array}$$

Variable range is

(22) $$\begin{array}{c} 0.05\leq x_{1}\leq 2.00,\\ 0.25\leq x_{2}\leq 1.30,\\ 2.00\leq x_{3}\leq 15.00. \end{array}$$

Results of HSMSSA for solving tension spring design problem are listed in Table 8, which are compared with SMA, SSA, AO, AOA, WOA, SCA, and MVO. It is evident that HSMSSA obtained the best results compared to all other algorithms.

#### 4.2.3. Three-Bar Truss Design Problem

**Figure 10:**
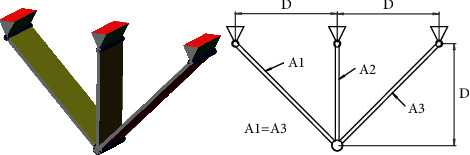
Three-bar truss design problem.

Three-bar truss design is a complex problem in the field of civil engineering [49]. The goal of this problem is to achieve the minimum weight in truss design.  Figure 10 shows the design of this problem. The formula of this problem can be described as follows.

Consider

(23) $$\begin{array}{c} \overset{⟶}{x}=\left[\begin{array}{cc} x_{1} & x_{2} \end{array}\right]=\left[\begin{array}{cc} A_{1} & A_{2} \end{array}\right]. \end{array}$$

Minimize

(24) $$\begin{array}{c} f\left(\overset{⟶}{x}\right)=\left(2\sqrt{2}x_{1}+x_{2}\right)*l, \end{array}$$

subject to

(25) $$\begin{array}{c} g_{1}\left(\overset{⟶}{x}\right)=\frac{\sqrt{2}x_{1}+x_{2}}{\sqrt{2}x_{1}^{2}+2x_{1}x_{2}}P-\sigma \leq 0,\\ g_{2}\left(\overset{⟶}{x}\right)=\frac{x_{2}}{\sqrt{2}x_{1}^{2}+2x_{1}x_{2}}P-\sigma \leq 0,\\ g_{3}\left(\overset{⟶}{x}\right)=\frac{1}{\sqrt{2}x_{2}+x_{1}}P-\sigma \leq 0. \end{array}$$

Variable range is

(26) $$\begin{array}{c} 0\leq x_{1},\\ x_{2}\leq 1, \end{array}$$

where *l*=100 cm, *P*=2 KN/cm^2^,  and *σ*=2 KN/cm^2^.

Results of HSMSSA for solving the three-bar design problem are listed in Table 9, which are compared with SMA, SSA, AO, AOA, WOA, SCA, and MVO. It can be observed that HSMSSA has an excellent ability to solve the problem in confined space.

#### 4.2.4. Speed Reducer Problem

**Figure 11:**
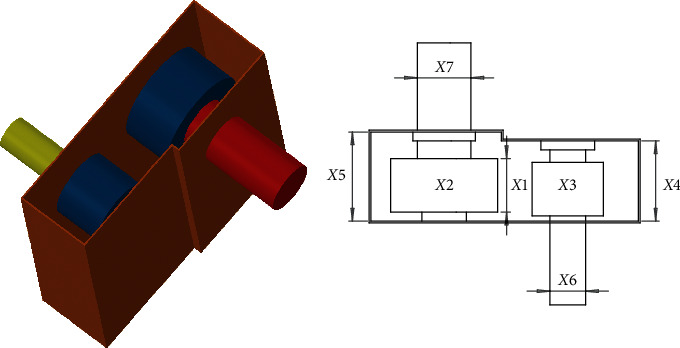
Speed reducer problem.

In this problem [15], the total weight of the reducer is minimized by optimizing seven variables.  Figure 11 shows the design of this problem, and the mathematical formula is as follows.

Minimize

(27) $$\begin{array}{c} f\left(\overset{⟶}{x}\right)=0.7854x_{1}x_{2}^{2}\left(3.3333x_{3}^{2}+14.9334x_{3}-43.0934\right)-1.508x_{1}\left(x_{6}^{2}+x_{7}^{2}\right)+7.4777\left(x_{6}^{3}+x_{7}^{3}\right), \end{array}$$

subject to

(28) $$\begin{array}{c} g_{1}\left(\overset{⟶}{x}\right)=\frac{27}{x_{1}x_{2}^{2}x_{3}}-1\leq 0,\\ g_{2}\left(\overset{⟶}{x}\right)=\frac{397.5}{x_{1}x_{2}^{2}x_{3}^{2}}-1\leq 0,\\ g_{3}\left(\overset{⟶}{x}\right)=\frac{1.93x_{4}^{3}}{x_{2}x_{3}x_{6}^{4}}-1\leq 0,\\ g_{4}\left(\overset{⟶}{x}\right)=\frac{1.93x_{5}^{3}}{x_{2}x_{3}x_{7}^{4}}-1\leq 0,\\ g_{5}\left(\overset{⟶}{x}\right)=\frac{\sqrt{{\left(745x_{4}/x_{2}x_{3}\right)}^{2}+16.9\times 10^{6}}}{110.0x_{6}^{3}}-1\leq 0,\\ g_{6}\left(\overset{⟶}{x}\right)=\frac{\sqrt{{\left(745x_{4}/x_{2}x_{3}\right)}^{2}+157.5\times 10^{6}}}{85.0x_{6}^{3}}-1\leq 0,\\ g_{7}\left(\overset{⟶}{x}\right)=\frac{x_{2}x_{3}}{40}-1\leq 0,\\ g_{8}\left(\overset{⟶}{x}\right)=\frac{5x_{2}}{x_{1}}-1\leq 0,\\ g_{9}\left(\overset{⟶}{x}\right)=\frac{x_{1}}{12x_{2}}-1\leq 0,\\ g_{10}\left(\overset{⟶}{x}\right)=\frac{1.5x_{6}+1.9}{x_{4}}-1\leq 0,\\ g_{11}\left(\overset{⟶}{x}\right)=\frac{1.1x_{7}+1.9}{x_{5}}-1\leq 0. \end{array}$$

Variable range is

(29) $$\begin{array}{c} 2.6\leq x_{1}\leq 3.6,\\ 0.7\leq x_{2}\leq 0.8,\\ 17\leq x_{3}\leq 28,\\ 7.3\leq x_{4}\leq 8.3,\\ 7.8\leq x_{5}\leq 8.3,\\ 2.9\leq x_{6}\leq 3.9,\\ 5.0\leq x_{7}\leq 5.5. \end{array}$$

The comparison results are listed in Table 10, which shows the advantage of HSMSSA in realizing the minimum total weight of the problem.

#### 4.2.5. Cantilever Beam Design

**Figure 12:**
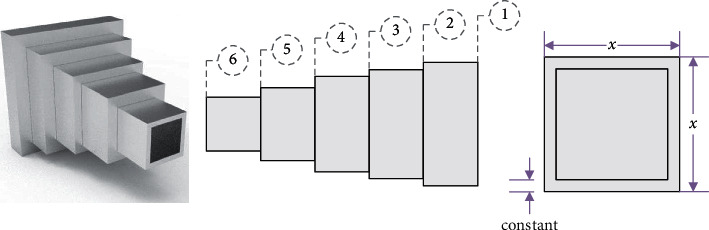
Cantilever beam design [24].

Cantilever beam design is a type of concrete engineering problem. This problem aims to determine the minimal total weight of the cantilever beam by optimizing the hollow square cross-section parameters [24].  Figure 12 illustrates the design of this problem, and the mathematical described is as follows.

Consider

(30) $$\begin{array}{c} x=\left[\begin{array}{ccccc} x_{1} & x_{2} & x_{3} & x_{4} & x_{5} \end{array}\right]. \end{array}$$

Minimize

(31) $$\begin{array}{c} f\left(\overset{⟶}{x}\right)=0.6224\left(x_{1}+x_{2}+x_{3}+x_{4}+x_{5}\right), \end{array}$$

subject to

(32) $$\begin{array}{c} g\left(\overset{⟶}{x}\right)=\frac{60}{x_{1}^{3}}+\frac{27}{x_{2}^{3}}+\frac{19}{x_{3}^{3}}+\frac{7}{x_{4}^{3}}+\frac{1}{x_{5}^{3}}-1\leq 0. \end{array}$$

Variable range is as follows: 0.01 ≤ *x*_1_, *x*_2_, *x*_3_, *x*_4_, *x*_5_ ≤ 100.

The results are shown in Table 11. From this table, we can see that the performance of HSMSSA is better than all other algorithms and the obtained total weight is minimized.

As a summary, this section demonstrates the superiority of the proposed HSMSSA algorithm in different characteristics and real case studies. HSMSSA is able to outperform the basic SMA and SSA and other well-known algorithms with very competitive results, which are derived from the robust exploration and exploitation capabilities of HSMSSA. Excellent performance in solving industrial engineering design problems indicates that HSMSSA can be widely used in real-world optimization problems.

## 5. Conclusion

In this paper, a Hybrid Slime Mould Salp Swarm Algorithm (HSMSSA) is proposed by combining the whole SMA as leaders and the exploitation phase of SSA as followers. At the same time, two strategies, including Levy flight and mutation opposition-based learning, are incorporated to enhance the capabilities of exploration and exploitation of HSMSSA. The 23 standard benchmark functions are utilized to evaluate this algorithm for analyzing its exploration, exploitation, and local optima avoidance capabilities. The experimental results show competitive advantages compared to other state-of-the-art metaheuristic algorithms, proving that HSMSSA has better performance than others. Five engineering design problems are solved as well to verify the superiority of the algorithm further, and the results are also very competitive with other metaheuristic algorithms.

The proposed HSMSSA can produce very effective results for complex benchmark functions and constrained engineering problems. In the future, HSMSSA can be applied to real-world optimization problems such as multiobjective problems, feature selection, multithresholding image segmentation, convolution neural network, or any problem that belongs to NP-complete or NP-hard problems.

## Figures and Tables

**Algorithm 1:**
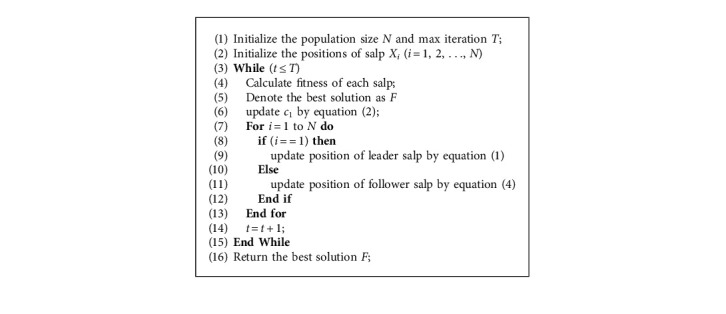
Pseudocode of Salp Swarm Algorithm.

**Algorithm 2:**
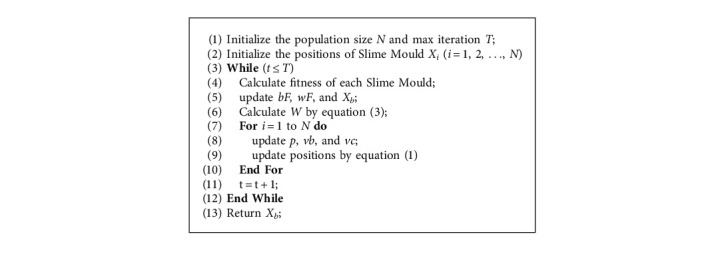
Pseudocode of Slime Mould Algorithm.

**Algorithm 3:**
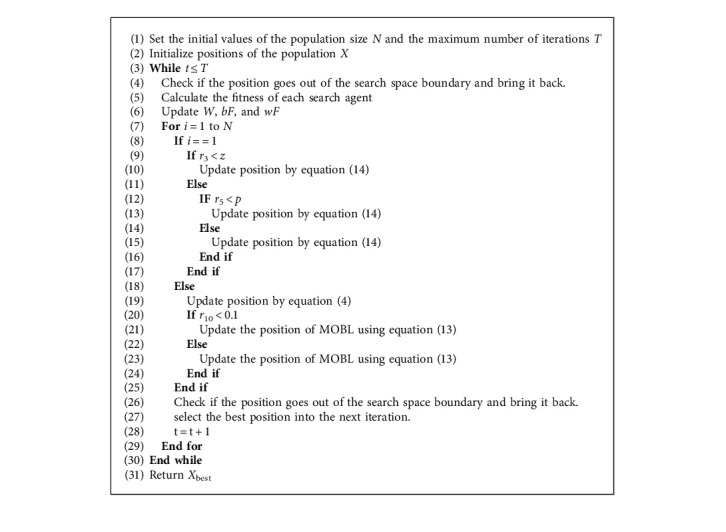
Pseudocode of HSMSSA.

**Table 1:** Unimodal benchmark functions.

Function	Dim	Range	*F*_min_
*F*_1_(*x*)=∑_*i*=1_^*n*^*x*_*i*_^2^	30	[−100, 100]	0
*F*_2_(*x*)=∑_*i*=1_^*n*^|*x*_*i*_|+∏_*i*=1_^*n*^|*x*_*i*_|	30	[−10, 10]	0
*F*_3_(*x*)=∑_*i*=1_^*n*^(∑_*j*−1_^*i*^*x*_*j*_)^2^	30	[−100, 100]	0
*F*_4_(*x*)=max_*i*_{|*x*_*i*_|, 1 ≤ *i* ≤ *n*}	30	[−100, 100]	0
*F*_5_(*x*)=∑_*i*=1_^*n*−1^[100(*x*_*i*+1_ − *x*_*i*_^2^)^2^+(*x*_*i*_ − 1)^2^]	30	[−30, 30]	0
*F*_6_(*x*)=∑_*i*=1_^*n*^(*x*_*i*_+5)^2^	30	[−100, 100]	0
*F*_7_(*x*)=∑_*i*=1_^*n*^*ix*_*i*_^4^+random[0,1)	30	[−1.28, 1.28]	0

**Table 2:** Multimodal benchmark functions.

Function	Dim	Range	*F*_min_
$F_{8}\left(x\right)=\sum_{i=1}^{n}-x_{i}\sin\left(\sqrt{\left|x_{i}\right|}\right)$	30	[−500, 500]	−418.9829 × Dim
*F*_9_(*x*)=∑_*i*=1_^*n*^[*x*_*i*_^2^ − 10 cos(2*πx*_*i*_)+10]	30	[−5.12, 5.12]	
$F_{10}\left(x\right)=-20\;\exp\left(-0.2\sqrt{\left(1/n\right)\sum_{i=1}^{n}x_{i}^{2}}\right)-\exp\left(\left(1/n\right)\sum_{i=1}^{n}\cos\left(2\pi x_{i}\right)\right)+20+e$	30	[−32, 32]	0
$F_{11}\left(x\right)=\left(1/4000\right)\sum_{i=1}^{n}x_{i}^{2}-\prod_{i=1}^{n}\cos\left(x_{i}/\sqrt{i}\right)+1$	30	[−600, 600]	0
$\begin{array}{c} F_{12}\left(x\right)=\left(\pi /n\right)\left\{10\;\sin\left(\pi y_{1}\right)+{\sum_{i=1}^{n-1}\left(y_{i}-1\right)}^{2}\left[1+10\;\sin^{2}\left(\pi y_{i+1}\right)\right]+{\left(y_{n}-1\right)}^{2}\right\}\\ +\sum_{i=1}^{n}u\left(x_{i},10,100,4\right),\text{where }y_{i}=1+\left(x_{i}+1\right)/4,\\ u\left(x_{i},a,k,m\right)=\left\{\begin{array}{cc} k{\left(x_{i}-a\right)}^{m}, & x_{i}>a,\\ 0, & -a<x_{i}<a,\\ k{\left(-x_{i}-a\right)}^{m}, & x_{i}<-a \end{array}\right. \end{array}$	30	[−50, 50]	0
$\begin{array}{c} F_{13}\left(x\right)=0.1\left(\sin^{2}\left(3\pi x_{1}\right)+\sum_{i=1}^{n}{\left(x_{i}-1\right)}^{2}\left[1+\;\sin^{2}\left(3\pi x_{i}+1\right)\right]\right)\\ +{\left(x_{n}-1\right)}^{2}\left[1+\;\sin^{2}\left(2\pi x_{n}\right)\right]\left(\right)+\sum_{i=1}^{n}u\left(x_{i},5,100,4\right) \end{array}$	30	[−50, 50]	0

**Table 3:** Fixed-dimension multimodal benchmark functions.

Function	Dim	Range	*F*_min_
*F*_14_(*x*)=((1/500)+∑_*j*=1_^25^1/(*j*+∑_*i*=1_^2^(*x*_*i*_ − *a*_*ij*_)^6^))^−1^	2	[−65, 65]	0.998
*F*_15_(*x*)=∑_*i*=1_^11^[*a*_*i*_ − (*x*_1_(*b*_*i*_^2^+*b*_*i*_*x*_2_)/(*b*_*i*_^2^+*b*_*i*_*x*_3_+*x*_4_))]^2^	4	[−5, 5]	0.00030
*F*_16_(*x*)=4*x*_1_^2^ − 2.1*x*_1_^4^+(1/3)*x*_1_^6^+*x*_1_*x*_2_ − 4*x*_2_^2^+*x*_2_^4^	2	[−5, 5]	−1.0316
*F*_17_(*x*)=(*x*_2_ − (5.1/4*π*^2^)*x*_1_^2^+(5/*π*)*x*_1_ − 6)^2^+10(1 − (1/8*π*))cos *x*_1_+10	2	[−5, 5]	0.398
$\begin{array}{c} F_{18}\left(x\right)=\left[\left[\right)1+{\left(x_{1}+x_{2}+1\right)}^{2}\left(19-14x_{1}+3x_{1}^{2}-14x_{2}+6x_{1}x_{2}+3x_{2}^{2}\right)\right]\\ \times \left[30+{\left(2x_{1}-3x_{2}\right)}^{2}\times \left(18-32x_{2}+12x_{1}^{2}+48x_{2}-36x_{1}x_{2}+27x_{2}^{2}\right)\right] \end{array}$	2	[−2, 2]	3
*F*_19_(*x*)=−∑_*i*=1_^4^*c*_*i*_exp(−∑_*j*=1_^3^*a*_*ij*_(*x*_*j*_ − *p*_*ij*_)^2^)	3	[−1, 2]	−3.86
*F*_20_(*x*)=−∑_*i*=1_^4^*c*_*i*_exp(−∑_*j*=1_^6^*a*_*ij*_(*x*_*j*_ − *p*_*ij*_)^2^)	6	[0, 1]	−3.32
*F*_21_(*x*)=−∑_*i*=1_^5^[(*X* − *a*_*i*_)(*X* − *a*_*i*_)^*T*^+*c*_*i*_]^−1^	4	[0, 10]	−10.1532
*F*_22_(*x*)=−∑_*i*=1_^7^[(*X* − *a*_*i*_)(*X* − *a*_*i*_)^*T*^+*c*_*i*_]^−1^	4	[0, 10]	−10.4028
*F*_23_(*x*)=−∑_*i*=1_^10^[(*X* − *a*_*i*_)(*X* − *a*_*i*_)^*T*^+*c*_*i*_]^−1^	4	[0, 10]	−10.5363

**Table 4:** Parameter settings for the comparative algorithms.

Algorithm	Parameters
SMA [35]	*z* = 0.03
SSA [27]	*c*_1_ = [1, 0]; *c*_2_ ∈ [0, 1]; *c*_3_ ∈ [0, 1]
AO [24]	*U* = 0.00565; *r*_1_ = 10; *ω* = 0.005; *α* = 0.1; *δ* = 0.1; *G*_1_ ∈ [−1, 1]; *G*_2_ = [2, 0]
AOA [15]	*α* = 5; *μ* = 0.5;
WOA [22]	*a*_1_ = [2, 0]; *a*_2_ = [−1, −2]; *b* = 1
SCA [14]	*a* = [2, 0]
MVO [10]	*WEP* ∈ [0.2, 1]; *TDR* ∈ [0, 1]; *r*_1_, *r*_2_, *r*_3_ ∈ [0, 1]

**Table 5:** Results of algorithms on 23 benchmark functions.

Function		HSMSSA	SMA	SSA	AO	AOA	WOA	SCA	MVO
F1	Mean	**0.0000E + 00**	3.3100*E* − 303	2.1200*E* − 07	3.4900*E* − 113	4.9600*E* − 06	3.0200*E* − 74	2.2039*E* + 01	1.2581*E* + 00
	Std	**0.0000E + 00**	**0.0000E + 00**	2.3600*E* − 07	1.9100*E* − 112	2.1600*E* − 06	1.6500*E* − 73	3.4334*E* + 01	4.4824*E* − 01

F2	Mean	**0.0000E + 00**	8.7800*E* − 161	1.9590*E* + 00	5.0900*E* − 59	1.9805*E* − 03	3.1700*E* − 50	2.6402*E* − 02	1.2927*E* + 00
	Std	**0.0000E + 00**	4.8000*E* − 160	1.4157*E* + 00	2.7900*E* − 58	1.9063*E* − 03	1.5800*E* − 49	2.9329*E* − 02	1.1310*E* + 00

F3	Mean	**0.0000E + 00**	1.4159e − 313	2.1110*E* + 03	3.7000*E* − 101	9.6539*E* − 04	4.3306*E* + 04	8.0776*E* + 03	2.3785*E* + 02
	Std	**0.0000E + 00**	**0.0000E + 00**	1.5514*E* + 03	2.0200*E* − 100	8.1952*E* − 04	1.6945*E* + 04	4.7486*E* + 03	1.2902*E* + 02

F4	Mean	**0.0000E + 00**	1.4800*E* − 150	1.1761*E* + 01	1.7300*E* − 55	1.7772*E* − 02	5.2341*E* + 01	3.4937*E* + 01	1.9521*E* + 00
	Std	**0.0000E + 00**	8.1300*E* − 150	4.0647*E* + 00	9.5000*E* − 55	1.1446*E* − 02	2.8502*E* + 01	1.3547*E* + 01	7.5824*E* − 01

F5	Mean	8.0753*E* − 02	5.0378*E* + 00	1.0827*E* + 02	**5.2141E − 03**	2.8034*E* + 01	2.8032*E* + 01	7.1843*E* + 04	4.7661*E* + 02
	Std	2.7660*E* − 01	9.1628*E* + 00	1.4901*E* + 02	**7.2117E − 03**	2.0336*E* − 01	4.1099*E* − 01	1.2854*E* + 05	7.8524*E* + 02

F6	Mean	**8.2900E − 07**	5.8093*E* − 03	1.3800*E* − 07	1.3859*E* − 04	3.1018*E* + 00	3.6069*E* − 01	1.1633*E* + 01	1.1004*E* + 00
	Std	**4.9600E − 07**	2.4730*E* − 03	1.5300*E* − 07	2.1774*E* − 04	2.2067*E* − 01	2.2358*E* − 01	9.7843*E* + 00	3.6012*E* − 01

F7	Mean	**6.4400E − 05**	1.6357*E* − 04	1.6394*E* − 01	7.8800*E* − 05	1.0695*E* − 04	3.0691*E* − 03	1.3682*E* − 01	3.6527*E* − 02
	Std	**6.2600E − 05**	1.3341*E* − 04	6.1309*E* − 02	6.5100*E* − 05	1.0095*E* − 04	2.8218*E* − 03	2.0698*E* − 01	1.6353*E* − 02

F8	Mean	**−1.2569E + 04**	−1.2569*E* + 04	−7.1073*E* + 03	−9.3742*E* + 03	−5.6142*E* + 03	−1.0050*E* + 04	−3.7684*E* + 03	−7.6853*E* + 03
	Std	**2.0511E − 02**	5.2108*E* − 01	9.0982*E* + 02	3.7928*E* + 03	3.9485*E* + 02	1.6846*E* + 03	2.8948*E* + 02	6.3161*E* + 02

F9	Mean	**0.0000E + 00**	**0.0000E + 00**	5.3927*E* + 01	**0.0000E + 00**	1.5400*E* − 06	**0.0000E + 00**	3.6516*E* + 01	1.2767*E* + 02
	Std	**0.0000E + 00**	**0.0000E + 00**	1.6886*E* + 01	**0.0000E + 00**	1.0800*E* − 06	**0.0000E + 00**	3.5214*E* + 01	3.5868*E* + 01

F10	Mean	**8.8800E − 16**	**8.8800E − 16**	2.5258*E* + 00	2.1600*E* − 14	4.2571*E* − 04	4.3200*E* − 15	1.4566*E* + 01	2.5576*E* + 00
	Std	**0.0000E + 00**	**0.0000E + 00**	6.8528*E* − 01	1.1400*E* − 13	1.8869*E* − 04	2.7200*E* − 15	8.3427*E* + 00	3.3443*E* + 00

F11	Mean	**0.0000E + 00**	**0.0000E + 00**	2.0677*E* − 02	**0.0000E + 00**	5.1653*E* − 04	1.9620*E* − 02	9.3797*E* − 01	8.7497*E* − 01
	Std	**0.0000E + 00**	**0.0000E + 00**	1.4808*E* − 02	**0.0000E + 00**	2.6954*E* − 03	5.4806*E* − 02	3.3652*E* − 01	7.4092*E* − 02

F12	Mean	2.4700*E* − 05	5.9758*E* − 03	7.1903*E* + 00	3.3600*E* − 06	7.3782*E* − 01	1.7457*E* − 02	1.4353*E* + 03	2.1818*E* + 00
	Std	5.1900*E* − 05	6.9167*E* − 03	2.7304*E* + 00	3.9400*E* − 06	2.7918*E* − 02	1.2428*E* − 02	7.0295*E* + 03	1.3736*E* + 00

F13	Mean	**4.6300E − 07**	5.4042*E* − 03	1.2368*E* + 01	1.4900*E* − 05	2.9623*E* + 00	5.1556*E* − 01	1.8722*E* + 05	1.4761*E* − 01
	Std	**1.4800E − 07**	7.9865*E* − 03	1.2274*E* + 01	2.0600*E* − 05	2.0518*E* − 02	2.3195*E* − 01	4.3886*E* + 05	6.2682*E* − 02

F14	Mean	**9.9800E − 01**	**9.9800E − 01**	1.2294*E* + 00	2.4408*E* + 00	1.0093*E* + 01	2.9945*E* + 00	1.7920*E* + 00	**9.9800E − 01**
	Std	**5.0400E − 16**	3.5900*E* − 13	6.7284*E* − 01	2.6435*E* + 00	3.9607*E* + 00	3.2280*E* + 00	9.8831*E* − 01	4.2300*E* − 11

F15	Mean	**3.9234E − 04**	5.4854*E* − 04	2.8300*E* − 03	4.8202*E* − 04	7.5731*E* − 03	8.4669*E* − 04	1.0574*E* − 03	5.7644*E* − 03
	Std	**1.0289E − 04**	2.5100*E* − 04	5.9501*E* − 03	1.0743*E* − 04	1.9781*E* − 02	6.5602*E* − 04	3.8453*E* − 04	1.2715*E* − 02

F16	Mean	**−1.0316E + 00**	**−1.0316E + 00**	**−1.0316E + 00**	−1.0311*E* + 00	**−1.0316E + 00**	**−1.0316E + 00**	**−1.0316E + 00**	**−1.0316E + 00**
	Std	**1.4300E − 13**	6.6000*E* − 10	2.3300*E* − 12	4.6089*E* − 04	2.7600*E* − 11	1.0400*E* − 09	4.2000*E* − 05	3.8000*E* − 07

F17	Mean	**3.9789E − 01**	**3.9789E − 01**	**3.9789E − 01**	3.9808*E* − 01	4.0051*E* − 01	**3.9789E − 01**	3.9921*E* − 01	**3.9789E − 01**
	Std	**2.6700E − 12**	3.0000*E* − 08	1.3400*E* − 10	2.2131*E* − 04	1.4391*E* − 02	6.9400*E* − 06	1.2200*E* − 03	1.3300*E* − 07

F18	Mean	3.9000*E* + 00	3.0000*E* + 00	**3.0000E + 00**	3.0365*E* + 00	1.2901*E* + 01	3.0003*E* + 00	3.0001*E* + 00	8.4000*E* + 00
	Std	4.9295*E* + 00	1.8000*E* − 10	**1.5000E − 13**	3.6597*E* − 02	2.1835*E* + 01	1.5988*E* − 03	9.1200*E* − 05	2.0550*E* + 01

F19	Mean	**−3.8628E + 00**	**−3.8628E + 00**	**−3.8628E + 00**	−3.8560*E* + 00	−3.7416*E* + 00	−3.8575*E* + 00	−3.8512*E* + 00	**−3.8628E + 00**
	Std	**4.2800E − 11**	8.4700*E* − 07	1.4600*E* − 10	7.4968*E* − 03	5.3652*E* − 01	5.7697*E* − 03	8.9408*E* − 03	1.9300*E* − 06

F20	Mean	**−3.2823E + 00**	−3.2426*E* + 00	−3.2259*E* + 00	−3.2075*E* + 00	−3.2903*E* + 00	−3.1996*E* + 00	−2.9186*E* + 00	−3.2655*E* + 00
	Std	**5.7048E − 02**	5.7100*E* − 02	5.9965*E* − 02	7.2462*E* − 02	6.3492*E* − 02	1.2887*E* − 01	3.7689*E* − 01	6.1457*E* − 02
F21	Mean	**−1.0153E + 01**	−1.0153*E* + 01	−6.3092*E* + 00	−1.0142*E* + 01	−7.4694*E* + 00	−8.1869*E* + 00	−2.4287*E* + 00	−7.0425*E* + 00
	Std	**1.0300E − 07**	1.9553*E* − 04	3.5233*E* + 00	2.6961*E* − 02	3.0153*E* + 00	2.6299*E* + 00	2.0334*E* + 00	3.0750*E* + 00

F22	Mean	**−1.0403E + 01**	−1.0403*E* + 01	−7.8362*E* + 00	−1.0392*E* + 01	−7.3418*E* + 00	−7.6682*E* + 00	−3.4206*E* + 00	−8.4065*E* + 00
	Std	**1.6600E − 07**	3.1185*E* − 04	3.4923*E* + 00	1.8669*E* − 02	3.2433*E* + 00	3.4333*E* + 00	1.7953*E* + 00	2.9406*E* + 00

F23	Mean	**−1.0536E + 01**	−1.0536*E* + 01	−9.4298*E* + 00	−1.0525*E* + 01	−7.9685*E* + 00	−7.2480*E* + 00	−3.6694*E* + 00	−9.6618*E* + 00
	Std	**9.5800E − 08**	2.5920*E* − 04	2.5607*E* + 00	1.6743*E* − 02	3.5174*E* + 00	3.2297*E* + 00	1.8159*E* + 00	2.3084*E* + 00

**Table 6:** The results of Wilcoxon's sign rank test for all functions.

Function	HSMSSA versus						
	SMA	SSA	AO	AOA	WOA	SCA	MVO
F1	**NaN**	6.1035*E* − 05	6.1035*E* − 05	6.1035*E* − 05	6.1035*E* − 05	6.1035*E* − 05	6.1035*E* − 05
F2	6.1035*E* − 05	6.1035*E* − 05	6.1035*E* − 05	6.1035*E* − 05	6.1035*E* − 05	6.1035*E* − 05	6.1035*E* − 05
F3	**NaN**	6.1035*E* − 05	6.1035*E* − 05	6.1035*E* − 05	6.1035*E* − 05	6.1035*E* − 05	6.1035*E* − 05
F4	6.1035*E* − 05	6.1035*E* − 05	6.1035*E* − 05	6.1035*E* − 05	6.1035*E* − 05	6.1035*E* − 05	6.1035*E* − 05
F5	6.1035*E* − 05	6.1035*E* − 05	4.2725*E* − 03	6.1035*E* − 05	6.1035*E* − 05	6.1035*E* − 05	6.1035*E* − 05
F6	6.1035*E* − 05	6.1035*E* − 05	6.1035*E* − 05	6.1035*E* − 05	6.1035*E* − 05	6.1035*E* − 05	6.1035*E* − 05
F7	2.1545*E* − 02	6.1035*E* − 05	**1.6882E − 01**	**7.1973E − 01**	1.8311*E* − 04	6.1035*E* − 05	6.1035*E* − 05
F8	6.1035*E* − 05	6.1035*E* − 05	6.1035*E* − 05	6.1035*E* − 05	6.1035*E* − 05	6.1035*E* − 05	6.1035*E* − 05
F9	**NaN**	6.1035*E* − 05	**NaN**	6.1035*E* − 05	**NaN**	6.1035*E* − 05	6.1035*E* − 05
F10	**NaN**	6.1035*E* − 05	**NaN**	6.1035*E* − 05	1.9531*E* − 03	6.1035*E* − 05	6.1035*E* − 05
F11	**NaN**	6.1035*E* − 05	**NaN**	6.1035*E* − 05	**NaN**	6.1035*E* − 05	6.1035*E* − 05
F12	6.1035*E* − 05	6.1035*E* − 05	3.0518*E* − 04	6.1035*E* − 05	6.1035*E* − 05	6.1035*E* − 05	6.1035*E* − 05
F13	6.1035*E* − 05	6.1035*E* − 05	3.0518*E* − 04	6.1035*E* − 05	6.1035*E* − 05	6.1035*E* − 05	6.1035*E* − 05
F14	6.1035*E* − 05	**5.0000E − 01**	6.1035*E* − 05	6.1035*E* − 05	6.1035*E* − 05	6.1035*E* − 05	6.1035*E* − 05
F15	1.5259*E* − 03	6.1035*E* − 05	1.2207*E* − 04	4.3721*E* − 02	1.8311*E* − 04	6.1035*E* − 05	1.2207*E* − 04
F16	6.1035*E* − 05	1.3184*E* − 02	6.1035*E* − 05	6.1035*E* − 05	6.1035*E* − 05	6.1035*E* − 05	6.1035*E* − 05
F17	6.1035*E* − 05	6.1035*E* − 05	6.1035*E* − 05	4.2725*E* − 04	6.1035*E* − 05	6.1035*E* − 05	6.1035*E* − 05
F18	**1.7334E − 01**	6.4697*E* − 03	4.3252*E* − 02	3.5339*E* − 02	4.3252*E* − 02	4.3252*E* − 02	4.3252*E* − 02
F19	6.1035*E* − 05	6.1035*E* − 05	6.1035*E* − 05	6.1035*E* − 05	6.1035*E* − 05	6.1035*E* − 05	6.1035*E* − 05
F20	2.5574*E* − 02	1.0254*E* − 02	1.1597*E* − 03	**2.2931E − 01**	4.5359*E* − 02	6.1035*E* − 05	**3.5913E − 01**
F21	6.1035*E* − 05	2.5574*E* − 02	6.1035*E* − 05	6.1035*E* − 05	6.1035*E* − 05	6.1035*E* − 05	6.1035*E* − 05
F22	6.1035*E* − 05	**4.2120E − 01**	6.1035*E* − 05	6.1035*E* − 05	6.1035*E* − 05	6.1035*E* − 05	6.1035*E* − 05
F23	6.1035*E* − 05	**4.2120E − 01**	6.1035*E* − 05	6.1035*E* − 05	6.1035*E* − 05	6.1035*E* − 05	6.1035*E* − 05
(W|L|T)	(17|1|5)	(20|3|0)	(19|1|3)	(21|2|0)	(21|0|2)	(23|0|0)	(22|1|0)

**Table 7:** Comparison of HSMSSA results with other competitors for the pressure vessel design problem.

Algorithm	Optimum variables				Optimum cost
	*T*_*s*_	*T*_*h*_	*R*	*L*	
**HSMSSA**	0.8533992	0.4183956	45.8059	135.5385	5961.5318
SMA	0.8744808	0.4273835	46.83887	125.5848	6008.4161
SSA	0.8934774	0.4387327	47.53594	119.2156	6047.6543
AO	0.8828092	0.4312524	47.26462	121.6449	6028.1585
AOA	0.807105	0.4426515	44.63354	147.5659	6052.8917
WOA	0.8310091	0.3646671	44.00895	154.3182	6047.0417
SCA	0.8820038	0.4335084	47.24144	125.7922	6139.5293
MVO	0.8380677	0.4992101	45.82367	135.3623	6253.5397

**Table 8:** Comparison of HSMSSA results with other competitors for the tension spring design problem.

Algorithm	Optimum variables			Optimum weight
	*d*	*D*	*N*	
**HSMSSA**	**0.05**	**0.348099**	**10.6486**	**0.011007**
SMA	0.057372	0.57232	4.1494	0.011584
SSA	0.05936	0.63542	3.4741	0.012256
AO	0.056912	0.55833	4.3271	0.011442
AOA	0.052579	0.41008	8.9843	0.012453
WOA	0.055292	0.47277	6.929	0.012906
SCA	0.0588	0.6172	3.65	0.012057
MVO	0.05251	0.37602	10.33513	0.012790

**Table 9:** Comparison of HSMSSA results with other competitors for the three-bar truss design problem.

Algorithm	Optimum variables		Optimum cost
	*x*_1_	*x*_2_	
**HSMSSA**	**0.78842**	**0.40811**	**263.8523**
SMA	0.79109	0.40022	263.8668
SSA	0.77823	0.43736	263.9363
AO	0.77899	0.43541	263.9197
AOA	0.76342	0.48382	264.3549
WOA	0.78357	0.42252	263.886
SCA	0.78058	0.43766	264.5463
MVO	0.75492	0.51216	264.7851

**Table 10:** Comparison of HSMSSA results with other competitors for the speed reducer design problem.

Algorithm	Optimum variables							Optimum weight
	*x*_1_	*x*_2_	*x*_3_	*x*_4_	*x*_5_	*x*_6_	*x*_7_	
**HSMSSA**	**3.4976**	**0.7**	**17**	**7.3**	**7.8**	**3.35006**	**5.28553**	**2995.4374**
SMA	3.51443	0.7	17	7.32444	7.80527	3.35235	5.28494	3002.5941
SSA	3.49767	0.7	17	7.87797	8.09401	3.3943	5.28574	3018.644
AO	3.5138	0.7	17	7.41461	7.81291	3.37709	5.28457	3009.9097
AOA	3.55989	0.7	17	7.49997	8.3	3.4623	5.28512	3061.8731
WOA	3.49739	0.7	17	7.87039	8.0769	3.45309	5.2854	3034.0596
SCA	3.6	0.7	17	7.3	8.3	3.37932	5.2799	3063.9102
MVO	3.6	0.7	17	8.3	8.3	3.38276	5.36041	3111.6609

**Table 11:** Comparison of HSMSSA results with other competitors for the cantilever beam design problem.

Algorithm	Optimum variables					Optimum weight
	*x*_1_	*x*_2_	*x*_3_	*x*_4_	*x*_5_	
**HSMSSA**	**5.9979**	**5.3289**	**4.4999**	**3.4817**	**2.166**	**1.3400**
SMA	6.0846	5.2309	4.5602	3.4725	2.1349	1.3405
SSA	6.1056	5.1001	4.303	3.7365	2.3183	1.3456
AO	5.8236	5.3813	4.5178	3.4768	2.3159	1.3426
AOA	5.7014	5.3722	4.6577	3.6643	2.1551	1.3448
WOA	5.8492	5.0587	4.8917	3.682	2.1564	1.3469
SCA	5.9165	5.9187	4.5133	3.3167	1.9823	1.3508
MVO	7.1088	5.0161	4.2203	3.4064	2.105	1.3647

## Data Availability

The data used to support the findings of this study are available from the corresponding author upon request.
